# Surgical Cases

**Published:** 1858

**Authors:** Wells R. Marsh

**Affiliations:** Prof. of Chemistry in Medical Department of Iowa University


### Cases in Surgical Practice.

BY WELLS R. MARSH, M. D.

(Prof. of Chemistry in Medical Department of Iowa University.)

### *Fracture of the Cervical Vertebræ—Recovery.*

CASE 1st—In a late No. of the New York Journal of Medicine, Dr. Daniel Ayres reports a case of dislocation of the cervical vertebræ, which he succeeded in reducing on the tenth day after the accident, with complete recovery of the patient. Very many instances have occurred, within the last few years, proving the fallacy of the popular, (and I might nearly as well have said professional) idea, of the necessarily fatal character of such injuries. An instance of fracture of one of the cervical vertebræ occurred, some three years since, in my own practice, which it may not be amiss to record, although I am uncertain if the course pursued in its treatment may be considered commendable or otherwise.

Mr. K——, a farmer, æt 43, large and athletic, in conveying a sack of wheat into a mill, tripped and fell with his head against another sack, the one upon his shoulder resting upon his neck. Being assisted to arise, he found his face turned toward the right shoulder, without the power of bringing it back to the natural position. He complained of pain in the neck upon every motion of the body. He, however, started in a wagon for home, twenty-four miles distant, made the journey, went to bed and applied warm fomentations to the neck, together with making use of domestic remedies, to produce a "sweat." The pain continuing to increase, he, on the succeeding morning, dispatched a messenger for me.

Upon my arrival, I found Mr. K. lying upon his back, with his head considerably raised and face turned to the right, at an angle, with the shoulder, of about twenty-five degrees. The posterior neck was much swollen and tender upon pressure. Pain was intense, and he could not move himself nor be moved without crying out. Could not change the position of the head—said that in attempting it, he "felt something rub, and heard a sound as if something was being filed." Upon raising him, the position of the head and neck in relation to the body remained the same. Placing one hand upon the head and the other upon the neck, and carefully attempting rotation of the head, I distinctly detected crepitus, and was unable to bring the face around beyond a certain point, where there was firm opposition, as if one solid body impinged against another. At about the articulation of the third with the fourth crevical vertebræ there appears to be a slight deformity—the direction of the spinous processes of these bones did not agree, but there was so much swelling I was unable to decide fully to my own satisfaction.

After careful examination, I concluded there was fracture of the left inferior articulating process of the third crevical vertebræ, or of the superior articulating process of the same side of the fourth.

I informed the patient, as dellicately as possible, that in my opinion he had broken his neck, when he desired me to set it and dress it in splints, and was hardly satisfied that I refused to do so. I was fearful that in manipulating to adjust the fracture, I should produce pressure upon the cord, and consequent fatal injury. I, consequently, simply arranged support for his head by bandages running to the shoulder; directed applications of cold water to the neck, with absolute rest, directing the patient to abstain from all experimental attempts to restore the natural position.

Following my direction, the patient slowly recovered, but, necessarily, with a deformity there is no possibility of remedying. It is less apparent than at first, however, since after the subsidence of all inflammation of the region, the rotary motion on the axis was regained, and the face could be brought around to nearly its natural position.

In this case I might have attempted a reduction of the fracture, and, perhaps, have succeeded without harm to the patient; but how to maintain the position, after adjustment, would then have been a question not easily decided. It was, on the whole, an ugly case, and I was only too well satisfied with the final result, without ever wishing I had attempted more.

Case 2nd.—W. V. S. a brick-mason, æt. twenty-three, called on me, on 18th July last to examine and prescribe for an inflamed foot. Said he had, the day previously, worn a tight new boot, which had caused considerable pain, but not sufficient to stop him from labor. Upon taking off the boot, in the evening, he found the foot considerably inflamed, and he passed a restless night. Morning found the inflammation greatly aggravated and he sent for me.

I found the foot very much swollen, and about the region of the tarso-metatarsal articulation there was redness, apparently of an erysipelatous character. Beneath, and to the inner side of the tarsal articulation of the metatarsal bone of the great toe, was a tubercle, discharging an oily matter. At this point there was distinct fluctuation, and I laid open the abscess by a free incision, when from two to three ounces of the oily substance was discharged. On the upper and outer surface of the foot, diagonally upward and outward from this point, I discovered a small, healthy cicatrix, and learned that it was caused by an accidental wound with a knife, twenty-one months before—that the knife was broken at the time, and the patient then thought the point was in the wound, but having had it examined by a physician, who could not detect the fragment, and as the wound healed slowly, but soundly, thereafter, he had concluded the blade had been lost, and, indeed, broken after the reception of the wound.

Several months afterward, after wearing a tight boot, the foot had been inflamed, somewhat as now, but had recovered again without suppurating, and he had been advised, by his physician, that the disease was then rheumatic.

Supposing the fragment of the knife to have been but the point of an ordinary penknife, I told the patient it was quite possible it was still retained, and was the cause of the present inflammation. Ordered poultices to the foot and rest.

The poultices were continued until the 22nd, when the patient discontinued them without my permission, as, he stated, "the foot was better, and he wanted it to have a chance to heal up." On the 23d I again visited him, and examining carefully the opening of the abcess made by the incision on the 18th, I discovered a black point at the bottom of the abcess, which was soon ascertained to be metallic. I siezed it with an ordinary pair of forceps, but was unable to move it from its position. I then procured a pair of strong plyers or pincers, such as are employed by smiths, and siezing the point with these, and exerting all the strength of my right arm, I drew out a piece of the blade of a dirk-knife, measuring one and seven-eighths inches in length, and three-fourths of an inch in its greatest breadth. The edge of the blade was broken out at one point—the surface was smooth and had not lost its metallic lustre.

The foot healed rapidly, and the patient says now "it is only a little weak."

The knife had been driven down, by the blow causing the wound, through the articulation between the cuboid and the external cuneiform bones, and had remained fast until the time of its extraction. The length of the fragment was so great, that it must have, from the first, protruded below these bones, the point resting in the soft tissues of the hollow of the foot.

It is no uncommon event for foreign bodies to be long retained in the body, without causing inconvenience, but it seems to me very remarkable, that one of such size and form should produce no disturbance in the position which this one occupied. Its being so securely fixed between the bones of the foot as to be entirely immoveable, while its point projected below, makes it appear almost impossible that irritation and inflammation should not have followed upon every attempt at locomotion.

This is, however, only one more fact which helps to prove that our philosophy is insufficient for the explanation of very many things, concerning which we think to understand so much.

